# Expanding the host range: infection of a reptilian host (*Furcifer pardalis*) by an atypical *Brucella* strain

**DOI:** 10.1007/s10482-020-01448-9

**Published:** 2020-07-22

**Authors:** Tobias Eisenberg, Karen Schlez, Ahmad Fawzy, Iris Völker, Silke Hechinger, Mersiha Curić, Nicole Schauerte, Christina Geiger, Jochen Blom, Holger C. Scholz

**Affiliations:** 1 Department of Veterinary Medicine, Hessian State Laboratory (LHL), Schubertstr. 60/ Haus 13, 35392 Giessen, Germany; 2 Department of Medicine and Infectious Diseases, Faculty of Veterinary Medicine, Cairo University, Giza Square, Giza, 12211 Egypt; 3 Frankfurt Zoo, Bernhard-Grzimek-Allee 1, 60316 Frankfurt, Germany; 4 Bioinformatics and Systems Biology, Justus-Liebig-University Giessen, Heinrich-Buff-Ring 58, 35392 Giessen, Germany; 5 Department of Bacteriology and Toxinology, Bundeswehr Institute of Microbiology, Neuherbergstrasse 11, 80937 Munich, Germany

**Keywords:** Brucella, Chameleon, Reptile, Cold-blooded animal, Poikilothermic, Whole genome sequencing, Devriesea agamarum

## Abstract

Atypical brucellae show deviant phenotypes and/or genotypes. Besides *Brucella inopinata*, *B. microti* and *B. vulpis*, atypical strains have been described infecting humans, rodents, amphibians and fish. They represent potential zoonotic agents. Here, we provide evidence that reptiles as the remaining poikilothermic vertebrate class also represent susceptible hosts for atypical *Brucella.*

**Electronic supplementary material:**

The online version of this article (10.1007/s10482-020-01448-9) contains supplementary material, which is available to authorized users.

## Introduction

The medically important genus *Brucella* comprises a historical clade of six so called classical *Brucella* species (the type species *Brucella melitensis* and *B. abortus, B. canis, B. ovis, B. neotomae* and *B. suis*) plus some recently described species including *B. pinnipedialis, B. ceti, B. microti, B. inopinata, B. papionis* and *B. vulpis* (Foster et al. 2007; Scholz et al. 2008, 2010, 2016b; Whatmore et al. 2014). A number of further atypical strains have been isolated in the past decade from humans, wildlife mammals, amphibians and fish (Eisenberg et al. 2012, 2017; Tiller et al. 2010a, b). Despite striking whole genome similarities of these monomorphic pathogens of above 99% atypical brucellae commonly carry additional genetic material not found in classical *Brucella* species but present in soil associated bacteria of the *Alphaproteobacteria* (Al Dahouk et al. 2017). Most of the accessory genes with known function encode additional metabolic proteins, ABC transporters or represent bacteriophages and mobile genetic elements that indicate a different ecology in comparison to the classical host-adapted *Brucella* species (Scholz et al. 2016a). To date, no such atypical members have been described infecting reptilian hosts, although this cold-blooded (poikilothermic) vertebrate class was hypothesized to be principally susceptible (Eisenberg et al. 2017; Mühldorfer et al. 2017). The present report focusses on the characterization of another novel member of the atypical *Brucella* group, recently isolated from the inner organs of a captive panther chameleon (*Furcifer pardalis*). To the best of our knowledge, this represents the first identification of a distinct *Brucella* sp. from a reptile host, thereby confirming principal susceptibility of these cold-blooded hosts.

## Material and methods

An adult 3-year old female panther chameleon (*Furcifer pardalis*) was submitted for necropsy following a history of weight-loss, swollen joints (both tarsi) and final euthanasia. Patho-histologically, tissue sections of multiple organs including liver, spleen, kidney, lung, gut, oviduct and tarsal joint were analyzed. Native tissue samples were processed for bacterial culture. Methods regarding bacterial culture, agglutination with anti-*Brucella* A- and M-antiserum, basic biochemical characterization with API 20NE (bioMeriéux, Nürtingen, Germany), matrix-assisted laser desorption/ionization-time of flight mass spectrometry (MALDI-TOF MS) analysis, *Brucella*-specific insertion sequence IS*711* PCR, have been previously described and published (Eisenberg et al. 2017) and are complemented here by modifications only: Subcultures were also incubated on brucella agar and in brucella broth with and without crystal violet under aerobic and capnophilic conditions at different incubation temperatures (20 and 37 °C). For MALDI-TOF MS measurements, isolates were prepared using a direct transfer protocol according to the manufacturer’s instructions (BrukerBiotyper, BrukerDaltonics, Bremen, Germany). A microflex LT system with MBT Compass software as well as the standard (DB 7854) and the security relevant (SR; both BrukerDaltonics) databases were used. The latter comprised spectra of 6 different *B. melitensis* strains, however, the database was further supplemented with quality controlled entries of atypical *Brucella* spp. from fish and frogs.

**Fig. 1:**
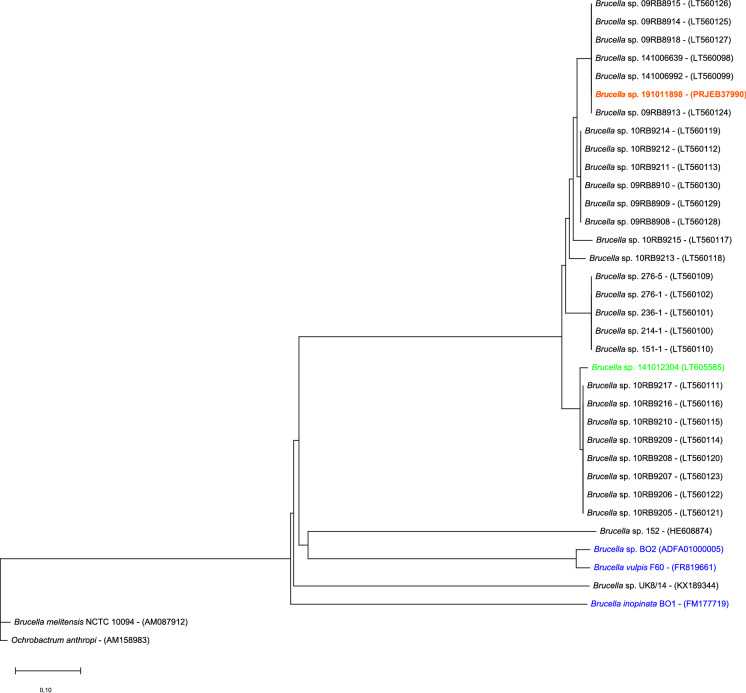
Phylogenetic tree based on partial RecA sequences (312 amino acids) of a dataset of atypical *Brucella* showing the phylogenetic position of strain 191011898. The tree was generated with MEGA X (Kumar et al. 2018) with the Neighbor-Joining method using standard settings. *Ochrobactrum anthropi* was used as outgroup; bar: 0.1 amino acids per site; isolation sources are indicated as follows: mammals (blue), reptile (red), amphibians (black), fish (green)

**Fig. 2:**
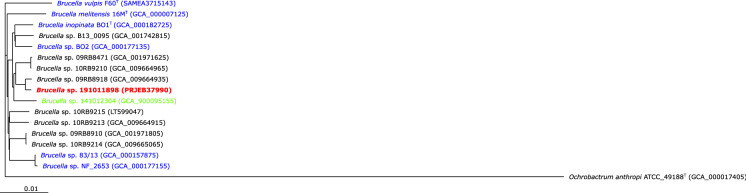
Core genome phylogenetic tree depicting strain 191011898 among other atypical *Brucella* strains. Core genes of these genomes were computed in EDGAR 2.0 (Blom et al. 2016) based on MUSCLE alignments and the Neighbor-Joining algorithm as implemented in the PHYLIP package. The core genome analysis was based on of 1,962 genes per genome in 17 *Brucella* species genomes (33,354 in total). The core has 647,357 amino acid residues/bp per genome, 11,005,069 in total. GenBank accession numbers are given in parentheses; bar, 0.01 nucleotide substitutions per site; isolation sources are indicated as follows: mammals (blue), reptile (red), amphibians (black), fish (green)

Amplification of the *Brucella*-specific insertion sequence IS*711* was done as described previously (Bricker and Halling 1994). The *recA* gene sequence was extracted from the genome sequence and compared with the consensus *recA* gene sequences of a representative classical brucella (*B. melitensis*) and members of the atypical group, comprising a set of 29 frog and one fish strains (Eisenberg et al. 2017; Scholz et al. 2016a), *B. inopinata* BO1^T^, strain BO2 and *B. vulpis* F60^T^. Briefly, partial RecA sequences (312 amino acids) were aligned using MUSCLE implemented in MEGA X (Kumar et al. 2018) with the Neighbor-Joining method using standard settings and 100 bootstrap repetitions. The type strain of *Ochrobactrum anthropi* served as outgroup (Fig. 1). The genome sequence was determined using the Illumina Hiseq (Illumina, Munich, Germany) sequencing platform and a Qiagen spin column kit (Qiagen, Hilden, Germany) for DNA purification (Eisenberg et al. 2017). Core genes of a representative set of atypical *Brucella* genomes were computed in EDGAR 2.0 (Blom et al. 2016) based on MUSCLE alignments and the Neighbor-Joining algorithm as implemented in the PHYLIP package (Fig. 2). Potential virulence factors were assessed using the VFanalyzer tool (https://www.mgc.ac.cn/cgi-bin/VFs/v5/main.cgi?func=VFanalyzer) available on the Virulence Factor Database (VFDB). The potential virulence factors were identified based on homologies with genomes of representative isolates of the genus *Brucella*, for which virulence factors are well characterized (Supplementary Table S1).

## Results

During *post mortem* examination of the adult female panther chameleon, a sanious coelomitis with approx. 3 ml of a brown exudate was found. Oviducts contained numerous multi-layered eggs and tarsal joints were affected by a bilateral septic arthritis. Patho-histologically, tissue sections of all organs contained multifocal areas of pyogranulomatous to necrotic inflammations with central necrosis and epitheloid macrophages that were surrounded by lympho-plasmacellular inflammation and calcification beside some protozoal stages. However, specific enrichment or staining for the presence of *Salmonella* spp. or *Cryptosporidium* spp. yielded negative results, but mixed Gram-positive and Gram-negative concomitant microbiota including *Vagococcus fluvialis*, *Staphylococcus sciuri* and *Pseudomonas aeruginosa* were isolated in moderate to high numbers from all tissues. Interestingly, a moderate growth of *Devriesea agamarum*, a primary reptilian pathogen, was also noted in moderate numbers from lung tissue (Martel et al. 2008). After two days of aerobic incubation on Columbia sheep blood and selective *Brucella* agar with and without crystal violet at 20 °C, smooth, convex, whitish colonies were isolated from all tissues that displayed a rapid cytochrome oxidase and urease reaction, but no motility. CO_2_ was not required for optimal growth. Cells were negative for agglutination with monospecific *Brucella* anti-M- and anti-A-serum and non-motile. Biochemical identification using the API20NE identification system yielded ‘*Ochrobactrum anthropi’* with an accordance of 99.9% (Bioprofile Number 1243364). The isolates were positive for nitrate reduction, urease, utilization of glucose, arabinose, mannose, *N*-acetyl glucosamine, maltose, adipic acid, malic acid and cytochrome oxidase. MALDI-TOF MS identified the reptile isolates as members of the genus *Brucella* with highest spectral similarity to the fish strain from the same zoo (Eisenberg et al. 2017). The genus affiliation was confirmed by IS*711* specific PCR.

One strain 191011898 from liver tissue was further investigated using whole genome sequencing (WGS; Acc. No. PRJEB37990). Based on both *recA* and core genome phylogenies the chameleon strain 191011898 from this study clustered most closely together with a group of strains obtained from amphibians from the same zoo (Figs. 1, 2). Furthermore, in a more in depth in silico analysis, homologs for common virulence genes found in classical *Brucella* spp. such as those expressing proteins involved in immune evasion and iron uptake were also demonstrated in strain 191011898 (Supplementary Table S1). Attempts to culture *Brucella* also from the chameleon’s and surrounding terraria did not yield any positive results following incubation of soil samples for up to 21 days at 20 and 37 °C on the above mentioned selective media with and without pre-enrichment in brucella broth.

## Discussion

Compared to classical *Brucella* spp. the group of atypical brucellae has an extended host spectrum besides mammals (including fish, amphibians and reptiles), may be metabolically active, fast growing and motile and thus is phenotypically and/or genotypically deviant (Mühldorfer et al. 2017; Scholz et al. 2016a). Since their first description in amphibians (Eisenberg et al. 2012) a worldwide distribution was found and our group has unravelled fish as the second vertebrate class to serve as a reservoir for members of the genus *Brucella* (Eisenberg et al. 2017). The results from this study now confirm our hypothesis that all three cold-blooded vertebrate classes are susceptible to *Brucella* infection. This picture is far less clear in birds, where occasionally anti-*Brucella* antibodies have been found, but more in depth studies are lacking, especially with respect to direct detection and clinical disease due to these microorganisms (Wareth et al. 2020). No natural *Brucella* infections have been described in adult birds to date with the exemption of one molecular proof in an avian blood sample of a migratory bird, but without any clinical signs (Najdenski et al. 2018; Wareth et al. 2020). However, since chicken embryos are susceptible and pathologies do occur depending on infection strains, brucellosis in birds needs further evaluation (Wareth et al. 2020).

The chameleon from this study was kept in the same zoo as some of our reported frogs (Eisenberg et al. 2012; Mühldorfer et al. 2017; Scholz et al. 2016a) and the bluespotted ribbontail ray (Eisenberg et al. 2017), but no known transmission routes (e.g. by feeder insects, supplies or equipment) were suspected and it was not possible to isolate the organism from the animal’s terrarium soil. The chameleon was captive-bred in another European zoo in 2016 and was received the same year with two unremarkable conspecifics for breeding purposes. Based on our findings (Fig. 1) more than 20 different *Brucella* isolates originating from the same zoo have been detected. Despite striking similarities between amphibian isolates and also to the most closely related *B. inopinata* (Figs. 1, 2), they do not represent clonal lineages. Contrarily, the *recA* phylogeny suggests that they belong to at least five different clusters. Because further amphibian isolates have been obtained also in other zoos and breeding facilities in Germany and abroad, we believe that these atypical *Brucella* strains do not share a common ancestor, but indeed originate from multiple, possibly environmental sources. The poly-bacterial findings in the case from this study do not unequivocally prove brucellae as the etiologic microorganism although they were isolated from all the tissues in high numbers and with patho-histological findings consistent with brucellosis. However, this case demonstrates that reptiles can in fact amplify and spread these harmful bacteria. Most atypical *Brucella* strains known to date and also the isolate from the present study have highest similarity to *B. inopinata*, isolated from a human, but deviant amphibian strains resembling *B. microti* have recently been found in frogs harvested for human consumption (Jay et al. 2018). With respect to discussion of zoonotic potential, there are a number of hints fostering the recommendation that atypical *Brucella* may represent serious human pathogens. Specifically, (1) strains BO1 and BO2 have been isolated from humans with brucellosis-like symptoms (De et al. 2008; Tiller et al. 2010b), (2) some strains (*B. microti*, BO1, Australian rodents) are lethal in the mouse model (Jiménez de Bagüés et al. 2014), (3) strains including isolates from amphibians were found to effectively multiply in different cell lines in vitro (Al Dahouk et al. 2017; Soler-Lloréns et al. 2016) and (4) most strains were capable to persist in mammalian hosts over a period of up to three months (Al Dahouk et al. 2017). Lastly, they (5) share identical virulence genes compared to classical brucellae (Al Dahouk et al. 2017; Soler-Lloréns et al. 2016) and (6) have been associated with significant morbidity and mortality in common voles and amphibians (Hubalek et al. 2007; Mühldorfer et al. 2017). On the other hand, one may argue that only weak pathological signs were observed in mice that cleared infections more efficiently and with fewer signs of inflammation compared to classical brucellae (Al Dahouk et al. 2017). Furthermore, no significant increase in human cases with brucellosis due to atypical brucellae is actually noted. Summarizing, since no direct transmission chains have been unravelled, yet, we cannot finally answer the question regarding the zoonotic potential, but we either cannot rule it out at present.

## Conclusions

This case highlights the principal significance of all three classes of cold-blooded vertebrate hosts, i.e. fish, amphibians and reptiles, with respect to and as a reservoir of *Brucella* infections with the potential of zoonotic transmission.

## Electronic supplementary material

Below is the link to the electronic supplementary material.

Supplementary file1 (XLSX 18 kb)

## Data Availability

All data have been made fully available to the public.
